# The Elusive Concept of Stability in Osteoporotic Vertebral Fractures: A Narrative Review

**DOI:** 10.3390/diagnostics16121896

**Published:** 2026-06-18

**Authors:** Nicolas Plais, Maria Isabel Almagro-Gil, Luis L. Urda, Luis Álvarez-Galovich, Mariana F. Fernández, José Luis Martín-Rodríguez

**Affiliations:** 1 Spine Unit, Hospital Universitario Clínico San Cecilio, 18007 Granada, Spain; maribelalmagrogil@gmail.com; 2 Trauma Unit, Hospital Universitario Clínico San Cecilio, 18007 Granada, Spain; luislopezurda@gmail.com; 3 Spine Unit, Hospital Universitario Fundación Jiménez Diaz, 28040 Madrid, Spain; lalvarez@fjd.es; 4 Instituto de Investigación Biosanitaria (ibs.GRANADA), 18012 Granada, Spain; marieta@ugr.es (M.F.F.);; 5 Centro de Investigación Biomédica, Universidad de Granada, 18016 Granada, Spain; 6 Ciber de Epidemiología y Salud Pública (CIBERESP), 28029 Madrid, Spain; 7 Radiology, Hospital Universitario Clínico San Cecilio, 18007 Granada, Spain

**Keywords:** osteoporosis, osteoporotic vertebral fractures, spinal instability, neurological deficits, vertebral body osteonecrosis, OVF classification

## Abstract

Osteoporotic vertebral fractures (OVFs) are the most common fragility fractures, representing a substantial burden on healthcare systems worldwide. Although up to 30% of OVFs may be clinically silent, a subset of patients experiences an unfavorable course, developing painful pseudoarthrosis/nonunion, progressive vertebral collapse, and even neurological compromise. While initial OVF management is typically nonoperative, a considerable proportion of patients ultimately require surgical intervention. However, clear and universally accepted surgical indications are lacking, rendering clinical decision-making complex and highly individualized. In this context, evaluating the spine’s ability to withstand physiological loads in the presence of potential instability is a critical step in the treatment algorithm. Nevertheless, spinal stability remains a dynamic and multifactorial concept that requires comprehensive assessment integrating both clinical and radiological parameters. This narrative review synthesizes the current state-of-the-art literature on the assessment of stability in OVFs, with particular clinical emphasis on clinical applicability. It revisits classical trauma-derived concepts and adapts them to the specific context of OVFs. We examine the respective roles of radiography, CT and MRI in evaluating fracture characteristics and spinal stability and summarize the main clinical and radiological markers. Furthermore, we distinguish between predictors of fracture progression and indirect indicators of established or evolving instability. Finally, we review current classification systems and outline general treatment considerations, focusing on how imaging findings may guide clinical decision-making in OVFs. Overall, this review provides a comprehensive framework of key imaging and clinical features that should be systematically assessed to estimate the risk of spinal instability.

## 1. Introduction

Osteoporotic vertebral fractures (OVFs) are among the most frequent fragility fractures, and their incidence rises markedly with age. They may account for at least 50% of the approximately 1.5 million osteoporotic fractures occurring annually in the United States [1]. Most OVFs are clinically silent, and only about 30% are identified at the time of injury [2,3]. Despite this underdiagnosis, the majority heal without sequelae [1,3]. However, in approximately 15–35% of patients, healing is incomplete, resulting in painful pseudoarthrosis or nonunion [1,3,4]. In addition, a substantial proportion of vertebral body fractures may progress to further collapse after mobilization [5], with progressive collapse reported in up to 30% in one series [4].

The impact on quality of life can be considerable, ranging from persistent pain and progressive kyphosis to severe loss of autonomy (e.g., difficulty standing or walking) and the onset of neurological symptoms [6,7]. Functional decline in patients with vertebral fractures has been reported to be comparable to that observed after hip fracture [8]. OVFs are also associated with an increased risk of subsequent vertebral fractures and increased mortality.

Stable fractures are usually managed conservatively with analgesia, early mobilization as tolerated, immobilization with or without a spinal orthosis, and treatment of underlying osteoporosis. In selected cases, percutaneous cement augmentation (vertebroplasty or kyphoplasty) may be considered. Nevertheless, management remains controversial: although management is often nonoperative, a substantial subset of patients ultimately require surgical intervention [9], and absolute indications are not consistently defined, underscoring the need for individualized decision-making [7].

This controversy highlights a key unmet clinical need: a structured and clinically applicable assessment of stability in OVFs. Spinal stability can be understood as the capacity to sustain physiological loads without progressive collapse and without the development of disabling pain, deformity, or neurological impairment. Assessing stability is, therefore, a critical step in guiding treatment decisions and predicting fracture evolution.

## 2. Search Strategy and Evidence Selection

This study was conducted as a narrative review and was not designed as a formal systematic review or meta-analysis. The objective was to provide a clinically oriented synthesis of the current evidence regarding instability in osteoporotic vertebral fractures (OVFs), with particular emphasis on imaging findings and their implications for clinical decision-making.

A literature search was performed using PubMed/MEDLINE, Web of Science, and Scopus, covering publications from January 2000 to December 2025. Search terms included combinations of keywords related to OVFs and instability, such as “osteoporotic vertebral fracture”, “spinal instability”, “vertebral collapse”, “posterior wall injury”, “nonunion”, “vertebral body osteonecrosis”, “kyphosis progression”, “neurological deficit”, “MRI”, “CT”, and “classification”.

Article selection was based on relevance, clinical applicability, and contribution to the understanding of instability in OVFs. Priority was given to studies evaluating predictors of fracture progression, instability, neurological compromise, nonunion, deformity progression, or treatment failure, as well as imaging-based analyses and treatment-oriented classifications. Seminal and widely cited publications were preferentially included because of their influence on current clinical practice. Exclusion criteria included studies not focused on osteoporotic or fragility vertebral fractures, purely technical reports without clinical or imaging correlation, and articles that could not be reliably interpreted. Additional references were identified through manual review of bibliographies from relevant articles. The principal studies supporting the proposed instability markers and decision framework are summarized in Supplementary Table S1.

Given the heterogeneity of the literature and the absence of universally accepted definitions of instability in OVFs, evidence was interpreted qualitatively. Greater weight was assigned to findings consistently reported across multiple studies and supported by imaging and clinical outcomes. When thresholds or definitions varied between studies, these were interpreted as reflecting the heterogeneity of the field rather than universally validated cut-offs. Consequently, the proposed framework should be regarded as a clinically oriented synthesis of the available evidence and expert interpretation rather than as a formal systematic review or meta-analysis.

## 3. Definition of Instability

Vertebral stability is a complex and multifactorial concept that remains difficult to define in clinical practice. White and Panjabi described clinical instability as a loss of the spine’s ability to maintain normal intervertebral relationships under physiological loads, such that this results in neurologic compromise, progressive deformity, and/or incapacitating pain [10]. In 1983, Denis introduced the three-column concept and proposed that instability typically reflects failure of at least two columns [11]; he also emphasized the key role of the middle column in thoracolumbar stability, suggesting that injuries with an intact middle column are usually stable. In 1990, White and Panjabi further operationalized the diagnosis by proposing a point-based checklist combining clinical findings and radiographic criteria; in this framework, a total score of ≥5 points suggests clinical instability [10]. Importantly, instability was not linked to any single pathognomonic feature: different constellations of findings could meet the threshold, underscoring that instability is multifactorial and context-dependent rather than a fixed entity.

Subsequent trauma classifications refined this approach by emphasizing injury morphology and the integrity of posterior stabilizing structures. Magerl highlighted distinct injury patterns [12], and later work (such as the TLICS framework described by Lee and Vaccaro and colleagues [13]) stressed the importance of posterior ligamentous complex (PLC) integrity.

These concepts are reflected in contemporary AO-based thoracolumbar classifications, which broadly converge on a practical principle: a fracture is generally considered stable when the posterior tension band/PLC is intact and the injury pattern is not expected to progress to deformity or neurologic compromise under physiological loading.

OVFs, however, occur in fragile bone and often exhibit patterns and failure mechanisms that differ from high-energy trauma; consequently, trauma-based classifications are not always directly applicable. Nevertheless, the central question remains the same: whether the spine (and the affected vertebra) can sustain physiological loads without progressive collapse, persistent pain, disability, or neurological deterioration. In OVF, “instability” is often less about immediate post-traumatic displacement and more about the risk of delayed progressive collapse and painful nonunion under everyday loading.

## 4. Imaging Techniques: How to Analyze Instability in OVF?

### 4.1. Radiography

Conventional radiography in two planes (AP and lateral) remains the first-line imaging modality to confirm an OVF. Although its sensitivity and precision are limited (particularly in early or subtle fractures) its wide availability, low cost, and ease of acquisition make it a fundamental part of the diagnostic work-up [14].

Full-spine radiographs are useful to identify synchronous or metachronous OVFs and to evaluate global spinal balance. When obtained standing (if tolerated), they provide a unique opportunity to assess sagittal alignment parameters, demonstrate sagittal or coronal malalignment, and help identify the main drivers of sagittal malalignment [15].

Beyond static views, functional radiographic techniques can contribute to the evaluation of instability. Flexion–extension radiographs may demonstrate dynamic instability and reducibility and can also support the diagnosis of painful nonunion/pseudoarthrosis when abnormal motion is seen at the index level [16]. Standing versus supine lateral radiographs can highlight posture-dependent changes (e.g., vertebral height loss or kyphotic angulation), thereby revealing abnormal motion at the fractured vertebra. Finally, supine hyperextension radiographs (e.g., on a bolster or block) can be helpful to assess reducibility and may assist treatment planning by demonstrating whether the deformity is correctable in extension [17].

### 4.2. Magnetic Resonance Imaging

MRI typically includes T1-weighted, T2-weighted, and a fluid-sensitive fat-suppressed sequence (most commonly STIR). In OVF, MRI is the reference standard for assessing fracture acuity, bone marrow and soft-tissue changes, and potential neural element compromise [14]. MRI contributes to the evaluation of OVF instability and differential diagnosis by enabling:

- Detection of additional vertebral fractures: It helps identify occult, synchronous, or metachronous vertebral body fractures that may be missed on plain radiographs.
- Assessment of PLC and posterior soft tissues: Edema or disruption of the interspinous/supraspinous ligaments is best appreciated on STIR sequences.
- Evaluation of neural elements and spinal canal compromise. When neurological symptoms are present (or when posterior wall involvement is suspected), MRI can identify neural injuries and causes of neurological compromise, including intramedullary hemorrhage, spinal cord contusion/edema, extrinsic compression (e.g., retropulsed fragments), traumatic disk herniation, and, rarely, complete spinal cord transection.
- Differentiation of acute/subacute versus chronic fractures: Acute or subacute OVF typically shows bone marrow edema (low signal on T1 and high signal on T2/STIR). Chronic/healed fractures usually lack marrow edema, helping distinguish new from old deformities and supporting clinical correlation.
- Differentiation between osteoporotic fracture and malignancy/infection: MRI can suggest alternative diagnoses when imaging features are atypical (e.g., diffuse marrow replacement patterns, soft-tissue mass, or other suspicious signal characteristics), and it helps guide the need for further evaluation (including contrast-enhanced MRI when appropriate).

### 4.3. Computed Tomography

CT is widely available, rapid, and highly accurate for characterizing OVFs, particularly when posterior wall involvement or compromise of the posterior bony elements is suspected [1]. Although CT is limited for assessing purely ligamentous injuries, it is excellent for evaluating the osseous structures that are critical to mechanical stability. CT is useful because it:

- Detects most fractures (reported detection rates of approximately 97–100%) while remaining less sensitive for purely ligamentous lesions.
- Provides superior bony definition, allowing for detailed assessment of the vertebral body as well as the pedicles and laminae.
- Is particularly helpful to identify retropulsion/intrusion of bony fragments into the spinal canal and to characterize translation or complex fracture morphology.
- Is often necessary to evaluate bony instability, especially when posterior wall disruption or posterior element fractures are present.
- Enables accurate measurement of spinal canal compromise (e.g., canal diameter/area) after injury, supporting assessment of neural risk and operative planning.

Extension CT has also been proposed as a functional technique. Guo et al. introduced an extended CT protocol obtained in the supine neutral position and in extension (typically prone with a firm pillow placed beneath the chest) [18]. This approach may help estimate fracture reducibility and potential recovery of vertebral height, information that can be relevant for treatment planning.

## 5. Instability Markers

Based on clinical signs and radiological parameters, instability markers can be identified. As discussed above, no single marker is sufficient to establish the diagnosis of instability. In OVF, presentations are heterogeneous and multiple fracture patterns may be encountered; therefore, an individualized assessment integrating symptoms, functional impact, and imaging findings is essential. Importantly, these markers do not all carry the same clinical weight and should be interpreted within the overall clinical context: some represent risk factors for future progression (e.g., collapse or nonunion), whereas others are indirect signs suggesting established or evolving instability. Rather than providing an absolute diagnosis on their own, they should be interpreted as warning signals along a spectrum. A thorough characterization of the fracture and its behavior under physiological loading is therefore critical to support appropriate decision-making.

### 5.1. Radiological Instability Markers

#### 5.1.1. Localization of the Vertebral Fracture

Fracture location influences the risk of instability and should be assessed systematically, particularly at the thoracolumbar junction, where the risk of progression is higher and where progressive collapse and poorer outcomes are most frequently reported [19,20,21]. Park et al. identified thoracolumbar junction involvement as a significant risk factor for delayed neurological compromise [22]. Tsujio et al. reported an increased risk of nonunion at this level [19], and Muratore et al. found that thoracolumbar localization was associated with failure of conservative treatment [21]. Biomechanically, this vulnerability may relate to the transition from the relatively rigid thoracic spine to the more mobile lumbar spine, which concentrates stresses at the junction. Similarly, Nardi et al. highlighted T12 and L1, as well as T7–T8, as “critical” vertebral levels [23], attributed to an increased flexion moment that may predispose these segments to deformity progression and instability.

#### 5.1.2. Conceptual Framework: Intravertebral vs. Intervertebral Instability

In this review, we propose a conceptual framework that distinguishes between intravertebral and intervertebral instability. We first address intravertebral instability, focusing on radiological features that reflect mechanical failure within the fractured vertebral body itself, considered independently of the adjacent motion segment. In the second part, we examine intervertebral (segmental/regional) instability, encompassing factors that reflect instability of a motion segment or a broader spinal region. This conceptual distinction, previously suggested in treatment-oriented studies of OVF nonunion [24,25], particularly by Patil et al. [25], is useful because it helps localize the presumed source of instability, whether primarily vertebral-body related or driven by segmental and regional biomechanics.

**A. Intravertebral instability**

**Figure 1:**
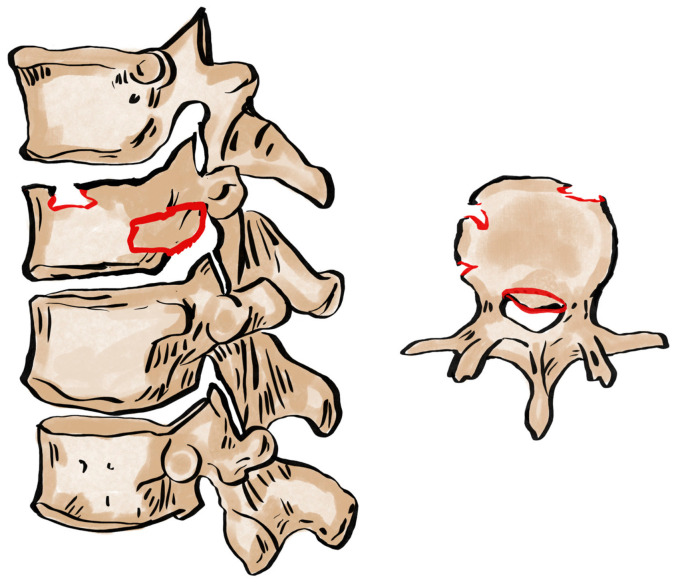
Illustration of intravertebral instability. The red lines indicate fracture lines involving the posterior wall and superior endplate, as well as retropulsed fragments migrated into the spinal canal.

In this section, we summarize vertebra-specific imaging markers (i.e., findings within the fractured vertebral body) that have been associated with delayed collapse, progressive deformity, nonunion/pseudoarthrosis, or persistent pain (Table 1 and Figure 1).

A decrease in segmental height is one of the most frequently reported intravertebral markers [26]. In the classical semiquantitative classification described by Genant et al. [27], a grade 3 deformity corresponds to a >40% reduction in vertebral height and is generally considered a severe fracture. Other authors have proposed that a reduction of >50% of the initial vertebral height predisposes patients to segmental instability and may increase the risk of subsequent OVFs [28]. Marked height loss may also upgrade severity within OVF-specific systems, reaching a severe category (e.g., OF4 in the DGOU classification) [29]. Moreover, in a multivariate analysis, Park et al. (2018) identified initial height loss as an independent risk factor for delayed neurological compromise [22]. Similarly, Viswanathan et al. identified an initial vertebral height loss of 25% on lateral radiographs as the ROC-derived cutoff associated with poor healing outcomes [30].

Posterior wall (middle column) injury is a key intravertebral marker because the middle column has a central role in thoracolumbar stability as already emphasized by Denis [11]. Hayashi et al. described how posterior wall fracture morphology influences posterior wall instability and spinal canal encroachment [31]. They proposed two patterns: a simple type and a comminuted type, the latter defined by posterior wall involvement in more than one fragment. The comminuted type was associated with greater canal encroachment under axial loading and, when surgery was required, tended to necessitate more rigid fixation. In line with this concept, both Guo et al. and Funayama et al. suggested that more severe posterior wall involvement is associated with instability [18,32]. Guo et al. considered posterior wall injury suggestive of instability when three or more fracture lines affect the posterior wall [18], whereas Funayama et al. emphasized the degree of canal encroachment, with Grade III injuries defined by encroachment ≥ 2 mm [32]. Finally, within OVF-specific frameworks, Spiegl et al. considered a complete burst fracture to correspond to an OF4 lesion, reflecting a higher-severity pattern with potential instability implications [33].

Although often consequent to posterior wall injury, spinal canal compromise is frequently reported as a radiological marker of fracture severity with potential implications for instability and neurological risk. Canal occupation by retropulsed bone fragments has been associated with an increased risk of neurological deficit, particularly when MRI shows a canal diameter reduction greater than 42% [6]. In cases with more severe canal compromise, especially exceeding 50%, more aggressive surgical management may be required [25].

Vertebral body shape has also been proposed as an imaging marker of instability. Since the lateral radiography-based classification described by Sugita et al. (2005) [34], morphological assessment has been incorporated into prognostic evaluation, with particular emphasis on anterior wall involvement. In their series, swelled-front, bow-shaped, and projecting types were associated with poorer outcomes, including a higher incidence of late collapse, and frequently demonstrated an intravertebral vacuum cleft. More recent studies have supported these observations: Muratore et al. reported a higher risk of progressive vertebral collapse in the swelling and bow-shaped patterns [20], while Tsujio et al. described an increased risk of nonunion in bow-shaped and projecting types [19].

Pedicle fractures are rare in OVF but are considered a marker of instability that may change the treatment indication and favor a more aggressive approach [18,32]. Funayama et al. further emphasized the increased likelihood of surgical treatment in the presence of pedicle fractures [32].

The presence of an intravertebral cleft has been reported as a risk factor for progressive vertebral collapse and an increased risk of neurological deficit [6,16,21]. Intravertebral clefts are fluid- or gas-filled linear, cystic, or nodular collections within a collapsed vertebral body. They may be detected on plain radiographs, on CT as intravertebral gas, or on MRI as a hyperintense (bright) area or a double line-sign on T2-weighted images with a hypointense (dark) or signal-void area on T1-weighted images [21].

Early reduction loss, a dynamic radiological parameter, has been linked to poorer outcomes. It reinforces the importance of close clinical and radiological surveillance, since changes over time are essential to the evaluation of instability. Spiegl et al. considered fractures unstable when loss of reduction exceeded 5° following mobilization [33].

Vertebral angular kyphosis, defined by an anterior-to-posterior vertebral body height ratio of less than 75%, is another important marker of intravertebral instability [35]. Takahashi et al. further reported that the anterior vertebral height ratio was significantly lower in the nonunion group than in the union group (75.8% vs. 84.5%, *p* < 0.001), suggesting that early anterior height loss may be associated with impaired fracture healing [36]. In cases of nonunion, Formica et al. classified this deformity as modifier B, as it may contribute to greater sagittal imbalance and require a more aggressive treatment strategy [35].

**Table 1:** Markers of intravertebral instability.

Marker	Modality	Treshold	Type	Clinical Implication
**Posterior wall/middle column injury**	CT scan	≥3 fracture lines [18]	Red flag	Suggests mechanical instability, consider surgery
**Canal compromise**	MRI	>42%–50% canal compromise associated with increased neurological risk [6,25]	Red flag	Increased risk of neurological deficits and possible need for surgical decompression
**Pedicle fracture**	CT Scan	Pedicle fracture, particularly bilateral involvement [18,32]	Red flag	Highly indicative of mechanical instability and possible surgical indication
**Angular dynamic instability > 15°**	Flexion-extension lateral radiographs	>15° increase in regional kyphosis on flexion-extension lateral radiographs [6].	Red flag	Suggests functional instability and increased neurological risk
**Posterior wall conminution**	CT Scan	Comminution, >1 posterior wall fragment [31], or canal encroachment ≥ 2 mm [32].	Major predictor of progression	Increased risk of failure of conservative treatment
**Initial vertebral height loss**	X-Rays	Height loss > 40% is considered indicative of a severe fracture [22,27,30].	Major predictor of progression	increased risk of delayed neurological symptoms
**Shape patterns**	X-Rays	Swelled-front, bow-shape, dented and projecting-types [19,20,34]	Major predictor of progression	Associated with increased risk of collapse and nonunion
**Intravertebral vacuum cleft**	MRI/CT Scan	Presence of intravertebral cleft/delayed healing	Major predictor of progression	Associated with nonunion, progressive collapse, and vertebral osteonecrosis
**Early reduction loss after mobilization**	X-Rays	Angular change > 5° after mobilization [33]	Major predictor of progression	Increased risk of failure of conservative treatment
**Vertebral Angular Kyphosis**	X-Rays	Anterior-to-posterior vertebral body height ratio < 75% [35]	Major predictor of progression	Associated with progressive deformity and possible need for anterior column restoration
**Positional vertebral mobility**	CT scan Standing X-Rays	≥6% difference in posterior vertebral height between standing radiographs and supine CT [37]	Major predictor of progression	Increased risk of vertebral colapse
**Increased density ratio > 2**	CT scan	HU density ratio ≥ 2 [37]	Emerging predictor	Potential predictor of progressive vertebral collapse
**Hinge-like fracture pattern**	Standing X-Rays	Severe imbalance between anterior vertebral body collapse and relative preservation of the posterior wall	Emerging predictor	Potential predictor of progressive kyphosis and sagittal malalignment

Hounsfield units (HU) values can be measured in the fractured vertebra and compared with those in an adjacent non-fractured vertebra. The density ratio is calculated by dividing the HU value of the non-fractured vertebra by that of the fractured vertebra. Interestingly, a density ratio ≥ 2 has been associated with an increased risk of vertebral collapse [37].

Dynamic fracture mobility is common in OVFs and represents one of the most clinically relevant indicators of instability in OVF. Based on a comparison of preoperative standing lateral radiographs with supine lateral radiographs, McKiernan identified mobility in up to 44% of patients [17]. Hoshino et al. defined angular instability as an increase of more than 15° in regional kyphosis on dynamic lateral radiographs (flexion/extension) and found that greater mobility was associated with back pain and more severe neurological deficits [6]. Moreover, Ruiz-Santiago et al. reported that a difference of at least 6% in posterior vertebral height (PVH) loss between standing CR and supine CT had an 88% discriminative power for predicting vertebral collapse [37].

Interestingly, Funayama et al. recently proposed a vertebral instability score integrating the change in vertebral collapse ratio on dynamic radiographs, the grade of posterior wall injury on MRI, and the presence of pedicle fracture on CT [32]. Higher scores were associated with a greater likelihood of surgical treatment, reinforcing the concept that instability reflects a combination of factors rather than a single sign. Okuwaki et al. further supported these findings, reporting that higher scores were also associated with an increased risk of progressive collapse [38].

In Table 1, the markers are classified according to their clinical relevance and current level of validation; however, these categories do not constitute a formal classification of the evidence.

**B. Intervertebral Instability/Regional Instability**

**Figure 2:**
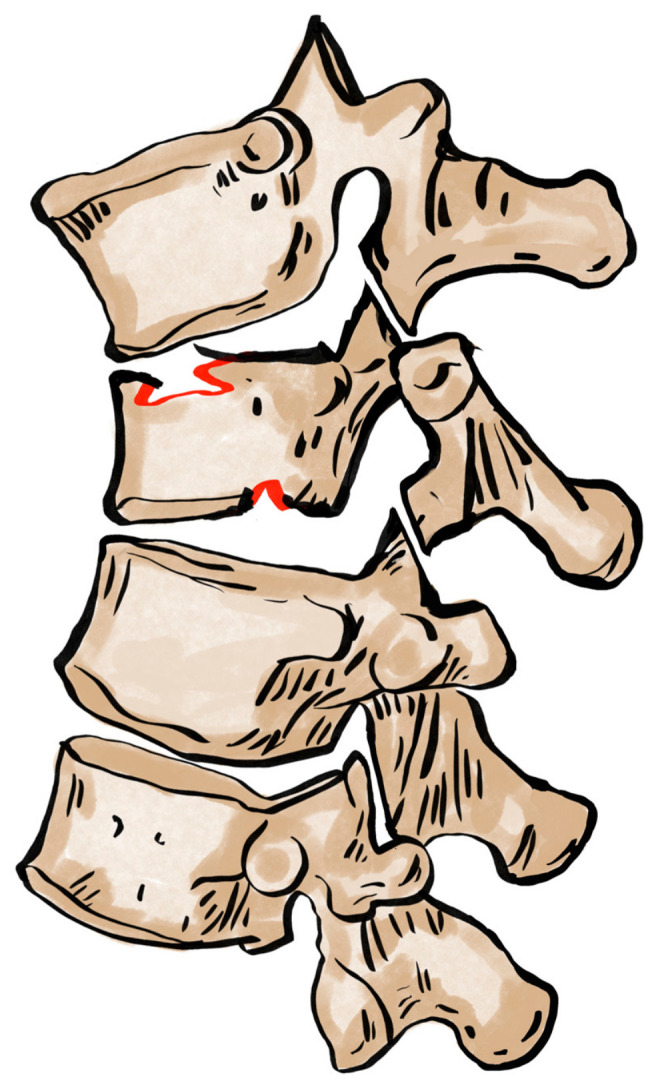
Illustration of intervertebral instability: The red lines indicate fracture lines involving both the superior and inferior endplates. Regional kyphosis is also observed.

The following section expands the analysis to intervertebral and regional instability, focusing on the structures that contribute to segmental stability. Beyond vertebral collapse itself, the main risks include the development of instability, kyphosis, malalignment, pain, and neurological deficit (Table 2 and Figure 2).

The most evident manifestation of segmental instability is spinal dislocation. These injuries result from displacement due to distraction and/or rotation and are classified as AO type C (or OF 5 in the OVF classification [39]). They are associated with major injury to key ligamentous stabilizers, frequently accompanied by fractures of the facet joints, lamina, posterior elements, and posterior ligamentous complex. Accordingly, they carry a high risk of neurological deficit, deformity, and chronic pain. However, these fracture patterns are uncommon in OVF; therefore, more subtle radiological indicators of intervertebral instability must be sought.

According to Chang et al. [40], degenerated disks together with their endplates represent some of the strongest components of the osteoporotic spine. However, endplate fracture lines or intervertebral disk injury can lead to significant translational instability at the disk–vertebral junction [25]. Moreover, dysfunctional intervertebral motion due to erosion of the adjacent endplate and disk degeneration may result in instability-related back pain, particularly after vertebroplasty [24]. Scheyerer et al. recently underscored the risk associated with fractures involving both endplates. They propose that these injuries ventralize the body’s center of gravity, thereby increasing torque above the fracture, paraspinal muscle demand, and compressive loading on adjacent endplates, resulting in a reported fivefold higher risk of adjacent vertebral fractures [41]. Finally, Seo et al. reported that OVFs involving both endplates are more prone to posterior wall collapse than fractures with single endplate involvement [42].

Assessment of the posterior ligamentous complex (PLC) is crucial in high-energy trauma and is a cornerstone of the TLICS scoring system [13]. The sequential ligament failure described by Pizones et al. illustrates how progressive disruption of the posterior stabilizers can lead to segmental instability with rupture of the supraspinous ligament (SSL) representing a clear-cut imaging sign of PLC incompetence [43]. Although less frequent, PLC injuries may also occur in OVF and should be carefully assessed. Guo et al. reported an association between PLC incompetence and neurological deficits [18], while Viswanathan et al. emphasized that PLC injury was one of the radiological factors most strongly associated with poorer outcomes and pseudarthrosis [30].

Increased regional kyphosis, measured as the angle between the superior endplate of the vertebra above and the inferior endplate of the vertebra below the fractured level, has long been considered a marker of segmental deformity and driver of surgical decision-making. In traumatic thoracolumbar burst fractures, Farcy et al. introduced the Sagittal Index to quantify segmental deformity and estimate the risk of late kyphotic progression [44]. In OVFs, regional kyphosis has similarly been associated with poorer outcomes [37]. Park et al. reported that patients who developed delayed neurological compromise had significantly greater initial kyphotic angle than those without neurological deterioration, supporting early kyphotic deformity as a risk marker rather than a definitive threshold. Although a kyphotic deformity exceeding 30° is often cited as a relevant surgical threshold [45,46], lower thresholds (>20º) have also been proposed by some authors [5]. Importantly, Patil et al. suggested that, in ununited osteoporotic vertebral compression fractures with neurological deficit, local kyphosis >30° may alter the surgical strategy, favoring more complex reconstructive procedures such as pedicle subtraction osteotomy rather than less extensive posterior decompression and fixation [25]. Therefore, regional kyphosis should be interpreted not only as a marker of deformity severity but also as a potential indicator of fracture chronicity, mechanical instability, and surgical complexity.

In Table 2, the markers are classified according to their clinical relevance and current level of validation; however, these categories do not constitute a formal classification of the evidence.

**Table 2:** Markers of Intervertebral instability.

Marker	Modality	Treshold	Type	Clinical Implication
**Displacement or dislocation**	**X-Rays**	OF 5/AO type C.	Red flag	High risk of neurological deficit and deformity; Usually favors surgical stabilization
**Major PLC Incompetence**	**MRI**	Major PLC injury [30,43]	Red flag	Suggests severe segmental instability
**Progressive regional kyphosis**	**Standing X-Rays**	Kyphosis > 20°–30° [5,45,46]	Major predictor of progression	Marker of deformity severity
**Disc-endplate injury**	**CT scan** **MRI**	Translational instability at the endplate-disc junction [25]	Major predictor of progression	Associated with instability-related pain
**Both endplates fractured**	**CT Scan** **MRI**	Present when both endplates are fractured [25,41,42]	Major predictor of progression	Iincreased risk of adjacent fractures and deformity progression
**Localization**	**X-Rays**	Thoracolumbar junction and transition zones [19,20,21]	Major predictor of progression	Increased risk of progression, instability, and nonunion
**Sagittal malalignment progression**	**Standing X-Rays**	Increased SVA/pelvic retroversion [15,47,48]	Major predictor of progression	Functional deterioration and increased risk of a domino-like vertebral fracture cascade

### 5.2. Clinical Markers of Instability

#### 5.2.1. Neurologic Symptoms

Acute neurological symptoms are generally considered a red flag for instability. According to Okuda et al. these may be related to several mechanisms, including direct neural compression by retropulsed bony fragments within the spinal canal, altered neural alignment secondary to progressive kyphosis, and dynamic neural injury caused by abnormal motion at the fracture site [49].

Patients with neurological involvement may present with weakness, sensory disturbances, radicular symptoms, and bowel or bladder dysfunction; in severe cases, they may develop cauda equina syndrome or complete spinal cord injury.

Delayed neurological symptoms are relatively frequent, occurring in up to 5.5% [22]. Instability at the fracture site appears to be the main contributing factor, leading to dynamic spinal cord compression [50]. In many case, the compression is related more to intervertebral disk injuries than to direct mechanical compression of the spinal cord by the bone fragments [50]. Other authors have identified additional risk factors for delayed neurological deterioration, including fractures at thoracolumbar junction, greater initial vertebral height loss, mid-portion fractures patterns, posterior wall involvement and fracture instability associated with intravertebral cleft (IVC) of the index vertebra [9,22,51].

#### 5.2.2. Persistent Severe Pain Despite Adequate Conservative Treatment

A substantial proportion of osteoporotic vertebral fractures are well tolerated, and nearly 30% are clinically silent. Pain is therefore not always present in OVF. For this reason, intractable pain (especially mechanical pain that worsens with standing or walking) should raise concern and may be an indirect sign of instability. In the DGOU/AO Spine decision framework described by Ullrich et al. persistent pain ≥ 4/10 on the VAS despite WHO step-2 analgesia is considered a warning feature within the OF score [52].

#### 5.2.3. Kyphosis and Sagittal Malignment

Focal kyphosis is a common feature of OVF. As discussed earlier, it may result from intravertebral instability (e.g., reduced anterior–posterior vertebral body height ratio) or from intervertebral instability (increased segmental kyphosis). A single anterior wedge fracture can increase thoracic kyphosis by 10° or more, and thoracic curves > 70° are frequent in older patients with multilevel compression fractures [53].

Kyphosis can cause pain at the fracture site, but it can also generate pain elsewhere by triggering compensatory mechanisms. Typical compensations include lumbar hyperlordosis (with increased facet loading), changes in thoracic curvature, and cervical hyperlordosis. If these mechanisms prove insufficient, compensatory changes shift distally, resulting in increased pelvic tilt and alterations in the hips and knees (hip extension and knee flexion). When compensatory capacity is exhausted, sagittal malalignment develops, characterized by increased sagittal vertical axis (SVA), increased Pelvic tilt and a forward-flexed posture. It is well established that sagittal malalignment has a major negative impact on quality of life [47].

Plais et al. reported that lumbar fractures and multiple fractures markedly increase the risk of sagittal malalignment [15]. OVF may act as an “accelerator” of imbalance: the combination of the degenerative cascade and fracture-related deformity can overwhelm compensatory reserve and lead to malalignment [15,54].

Importantly, kyphosis and sagittal malalignment are not only consequences of OVF but may also promote new fractures by altering alignment and load distribution [55]. Increased kyphosis shifts the load anteriorly on the vertebral body [5]. Nardi et al. noted that increasing kyphosis raises the flexion moment, a phenomenon often described as a “domino effect” [23]. Similarly, Alexandru et al. suggested that when one segment collapses to the point of instability, adjacent levels must bear additional load, which may contribute to degeneration and/or further vertebral compression fractures [28]. Sagittal malalignment has also been associated with a higher risk of nonunion [48].

For these reasons, treatment planning should include a careful assessment of deformity and sagittal alignment. Formica et al. emphasized the role of deformity and sagittal malalignment in decision-making, and SVA is the second modifier in their treatment-oriented classification for vertebral body osteonecrosis [35].

#### 5.2.4. Hinge-like Fracture Pattern: A Hypothesis-Generating Clinical Observation

**Figure 3:**
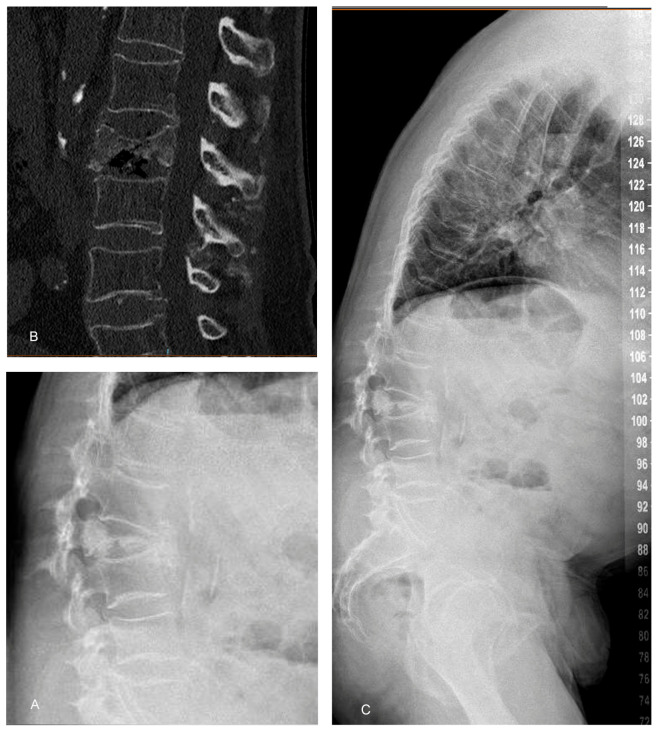
Seventy-seven-year-old man with an L2 fracture and severe sagittal malalignment. (**A**) Plain radiographs demonstrating segmental kyphosis related to marked anterior column collapse, while the relatively preserved posterior wall acts as a hinge-like structure. (**B**) CT scan showing intravertebral vacuum cleft consistent with Kümmell disease. (**C**) Standing radiographs demonstrating severe global sagittal malalignment.

**Figure 4:**
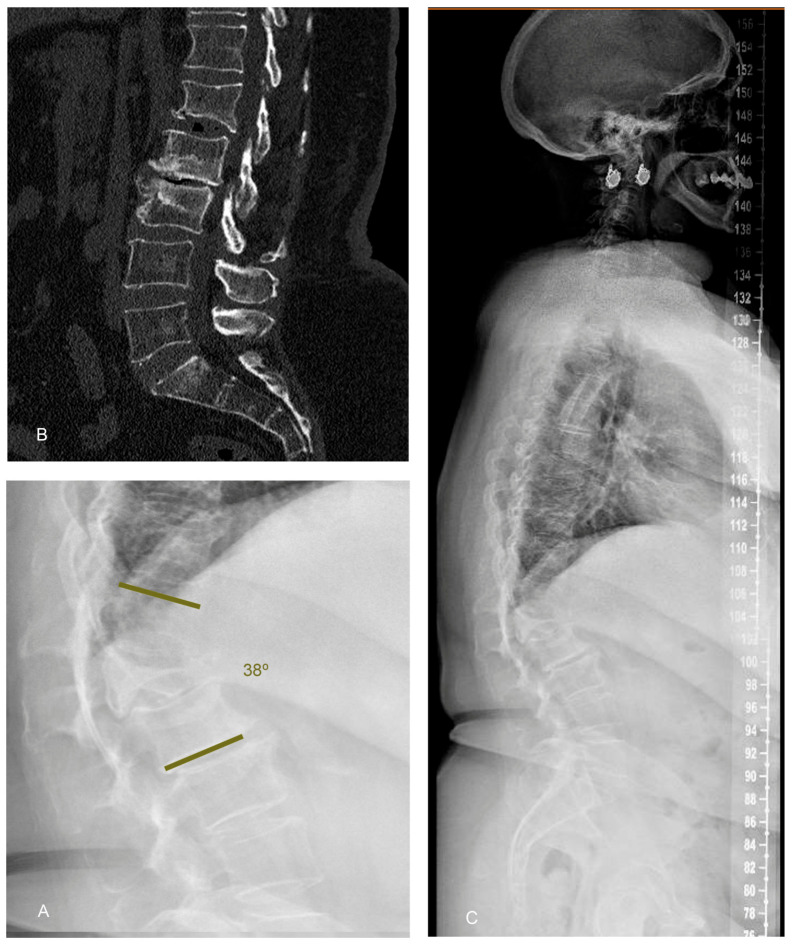
Seventy-year-old woman with an L3 fracture and relative preservation of the posterior wall. (**A**) Plain radiographs demonstrating regional kyphosis of 38°, caused by collapse of the anterior column while the relatively preserved posterior wall acts as a hinge-like structure. (**B**) CT scan showing fracture involvement of the inferior endplate. (**C**) Standing radiographs demonstrating the absence of major global sagittal malalignment due to compensatory mechanisms.

We would like to highlight a fracture pattern that we have frequently observed and propose as a potential morphological configuration associated with an increased risk of progressive kyphosis and sagittal malalignment. These fractures are characterized by near-complete collapse of the anterior column, while the posterior wall remains relatively preserved. In some cases, a marked reduction in the anterior-to-posterior vertebral height may contribute to pronounced local kyphosis. As the anterior portion of the vertebral body consolidates in a shortened, wedge-shaped configuration, progressive anterior spinal angulation may occur. In this setting, the relatively intact posterior wall may act as a hinge-like structure, facilitating further kyphotic progression and contributing to global sagittal imbalance (Figure 3 and Figure 4). However, this concept should be interpreted as a hypothesis-generating observation derived from clinical experience and illustrative cases, rather than as a validated fracture subtype, and therefore requires further investigation and validation.

### 5.3. New Horizons: Artificial Intelligence and Radiomics

In the era of artificial intelligence (AI), machine learning (ML) and deep learning (DL) approaches are increasingly being applied to the diagnosis and prognosis of OVFs. These techniques enable the automated extraction of imaging patterns and may support data-driven clinical decision-making. Radiomics, often combined with AI methods, is emerging as a transformative tool in radiology [56]. It involves converting into quantitative data: once a vertebra has been segmented on radiographs, CT, or MRI, specialized software extracts numerous features describing shape, intensity, and texture, which are then selected and integrated to develop diagnostic or prognostic models. Together, these approaches may improve the diagnosis and management of OVFs by enhancing diagnostic accuracy, fracture risk prediction, and the development of personalized treatment strategies [57].

Current evidence suggests three main areas in which AI may be useful in OVFs. First, AI can support screening and diagnosis, as several studies have shown accurate detection of OVFs on plain radiographs [58,59], MRI [60], and CT [61]. Improved opportunistic detection may facilitate earlier treatment and closer follow-up of fractures that might otherwise be overlooked. Second, AI may enhance fracture classification and risk stratification. Gu et al. summarized studies in which DL models, including GoogLeNet, Xception, ResNet, and 3D-ResNet, were used to classify vertebral fractures according to type or severity [62]. In another study, Zhang et al., applied the MagNet architecture to the diagnosis of vertebral fractures and three-column injury [63]. Additionally, AI may help differentiate OVFs from fractures of malignant origin [64]. Third, AI may help predict clinical outcomes, including the risk of future fractures and recurrent postoperative fractures. In a systematic review of 13 studies including 24,489 patients, AI models showed high overall performance in fracture prediction and appeared to outperform DXA and FRAX, although larger multicenter studies are still needed before routine clinical implementation [65].

In the near future, AI-based models may also help identify unstable fractures and those most likely to require active treatment. However, most current approaches rely on supervised or semi-supervised learning and therefore depend on high-quality ground truth labels, highlighting the need for standardized diagnostic criteria for unstable OVFs within the research community. Importantly, current AI systems remain limited by their inability to capture dynamic biomechanical information, in contrast to functional radiography, which may restrict their utility in assessing fracture instability. A more in-depth analysis of current trends and applications of AI in OVFs is beyond the scope of this review.

## 6. Specific Factors and Progression of Instability

### 6.1. Patient Specific Risk Factors

In addition to the clinical and radiological characteristics of each fracture, several authors have identified patient-specific risk factors for instability and poorer outcomes. Older age, severe osteoporosis, and overweight status (BMI > 25.5 kg/m^2^) have been reported as predictors of conservative treatment failure [41,66].

Hoshino et al. underscored the risk associated with prior steroid use, whereas Okuwaki et al. emphasized the association between low 25(OH)D levels at admission and an increased risk of OVFs [6,38]. Fat infiltration and reduced muscle mass of the erector spinae on MRI [67] have also been associated with a higher risk of OVFs, while reduced cross-sectional area (CSA) of the psoas has been linked to increased local kyphosis [38]. Moreover, Kusukawa et al. identified severe fatty degeneration of the paraspinal muscles as an independent risk factor for domino OVFs [67]. These factors underscore the importance of biological status and frailty in the overall assessment of the patient, as well as in guiding both the selection and timing of the treatments that the patient is able to tolerate.

### 6.2. Acute vs. Delayed Instability

We define acute instability when there is a clear timeline for the traumatic event and the initial assessment of the fracture shows obvious signs of instability. In contrast, delayed instability refers to cases in which the initial assessment suggests a stable fracture, but follow-up demonstrates progression toward instability.

Spiegl et al. described several mechanisms that may explain why an initially stable fracture can become unstable [5]. Repetitive loading of the fractured vertebral body may lead to posterior wall insufficiency and progressive regional kyphosis through a “kyphotic cascade.” In this process, nonphysiological force vectors promote degenerative disk changes and loss of disk height, thereby increasing peak loads on the vertebral body, both anteriorly and posteriorly. Progressive weakening of the bony structure then leads to further kyphosis and/or collapse.

### 6.3. Non Union and Vertebral Body Osteonecrosis (VBON)

Nonunion and vertebral body osteonecrosis (VBON, Kümmell disease) are two related entities associated with delayed instability after OVF. Both are considered late complications, and the distinction between them is often blurred, with overlapping clinical and imaging findings [35].

Nonunion refers to the failure of the fracture healing for several months (often discussed clinically when consolidation does not progress and symptoms persist). VBON/Kümmell disease, first described by Hermann Kümmell in 1891, classically presents as a delayed vertebral body collapse with progressive painful kyphosis after a pain-free interval, which is thought to be related to ischemia/microvascular compromise of the vertebral body [66,68]. Previous studies report delayed union/nonunion rates of 13.5–19.6% in OVF at 6-month follow-up [69].

Although vertebral biopsy is the diagnostic gold standard for osteonecrosis, diagnosis in practice is usually guided by the clinical course plus imaging [68]. A key imaging feature is the intravertebral vacuum cleft, i.e., gas or fluid within the collapsed vertebral body (sometimes visible on plain radiographs but typically easier on CT); a linear cleft morphology is strongly associated with benign ischemic necrosis. On MRI, the cleft may appear as fluid (low signal on T1, high on T2) and the double-line sign on T2-weighted sequences, an inner bright line with an outer dark rim around the necrotic/cleft area, is considered highly indicative of VBON [25,68,70].

Tsujio et al. identified the following risk factors for chronic pseudarthrosis/VBON: fractures at the thoracolumbar junction, middle-column injury (suggesting a burst component), and specific T2-weighted MRI patterns, either a confined high-intensity area or a diffuse low-intensity area within the fractured vertebra, each associated with a higher risk of OVF nonunion [19].

Instability related to VBON may lead to back pain, progressive kyphosis, vertebral collapse, and an increased risk of neurological deficits [6,51,70]. Hoshino et al. described two imaging patterns associated with a higher likelihood of neurological symptoms: angular instability, defined as a change in regional kyphosis of more than 15° on flexion–extension lateral radiographs, and severe spinal canal compromise, defined as more than 42% canal occupation by retropulsed bony fragments [6].

## 7. Classifications

As mentioned above, trauma-based classifications are not optimal for OVF, and several authors have proposed systems specifically designed to support diagnosis and treatment planning.

Based on lateral plain radiographs, Genant et al. graded OVF according to vertebral height loss and the site of deformity (anterior, middle, and/or posterior vertebral body) [27]. A normal vertebra is grade 0; mild deformity (<25% height loss) is grade 1; moderate deformity (25–40%) is grade 2; and severe deformity (>40%) is grade 3. The system showed substantial interobserver reliability and was widely used as a diagnostic and prognostic tool. However, it did not address distraction injuries or displaced unstable fractures, which limited its utility for surgical decision-making.

In 2005, Sugita et al. proposed a shape-based classification on lateral radiographs, describing five patterns [34]: swelled-front type (swelling of the anterior wall), bow-shaped type (pinched anterior wall with endplate collapse, resembling a ship’s bow), projecting type (anterior wall projection appearing as a small bulge without a clear fracture line), concave type (endplate collapse with an intact anterior wall), and dented type (central denting of the anterior wall with a visible fracture line). Sugita reported that the swelled-front, bow-shaped, and projecting types were associated with a worse prognosis, including a higher rate of late collapse and frequent vacuum cleft formation.

**Figure 5:**
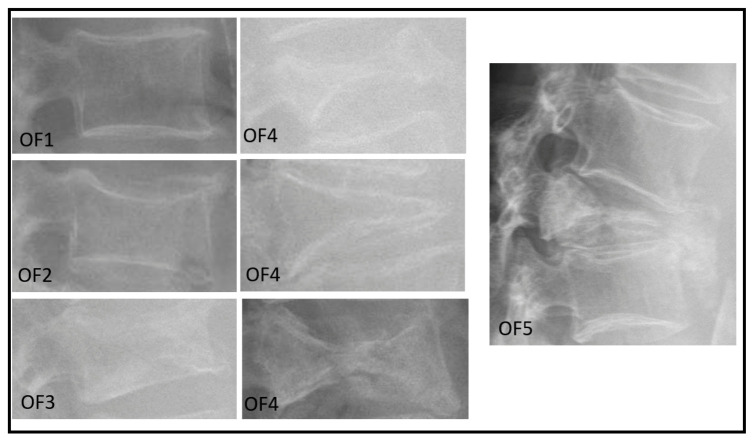
Morphological patterns of the AO Spine–DGOU Osteoporotic Fracture (OF) classification.

The Schnake classification (2018), later referred to as the AO Spine–DGOU Osteoporotic Fracture (OF) classification [29], is one of the most useful systems for OVF. It incorporates typical morphological patterns as well as the biomechanical stability of the fracture (Figure 5). Fractures are divided into five categories: OF 1, no vertebral deformity (bone marrow edema on MRI-STIR only); OF 2, deformity with no or only minor posterior wall involvement (<1/5); OF 3, deformity with distinct posterior wall involvement (>1/5); OF 4, loss of vertebral frame structure, vertebral body collapse, or a pincer-type fracture; and OF 5, injuries with distraction and/or rotation. A recent study showed that the OF classification is suitable for grading fracture severity and that higher OF grades correlate with greater surgical invasiveness [39]. The DGOU classification has several limitations. It does not take into account that height loss or deformities may be secondary to causes other than fractures, nor does it account for the impact of osteoporotic vertebral fractures on sagittal alignment [71]. Furthermore, it does not provide guidance for treatment in patients with multiple fractures, a common clinical scenario.

Of particular interest for its treatment-oriented approach, the classification proposed by Formica et al. for VBON defines four stages based on flexibility and disease progression: stage 0 (theoretical phase), stage 1 (early phase), stage 2 (instability phase), and stage 3 (fixed deformity phase). Treatment is further guided by two modifiers: angular kyphosis and global sagittal alignment (it is the first classification to take into account the global spine) [35].

## 8. What Comes Next After the Diagnosis

In all patients, anti-osteoporotic therapy is essential. Preventing new fractures is often as important as treating the index fracture, since the risk of subsequent fractures increases sharply after a first OVF [72]. Untreated osteoporosis is associated with recurrent fractures, leading to persistent pain, progressive kyphosis, sagittal malalignment, and loss of independence.

Conservative management remains the standard for stable fractures. Cement augmentation procedures (e.g., vertebroplasty/kyphoplasty) may reduce pain and can partially restore vertebral height when performed early, but indications remain controversial [55,73] and are beyond the scope of this review.

### 8.1. Surgical Indications for Unstable Fractures

Surgical indications in OVF remain a matter of debate. There is broad consensus that surgery is indicated in the presence of acute or delayed neurological deficits [9,25,26,40,50,66,74].

**Figure 6:**
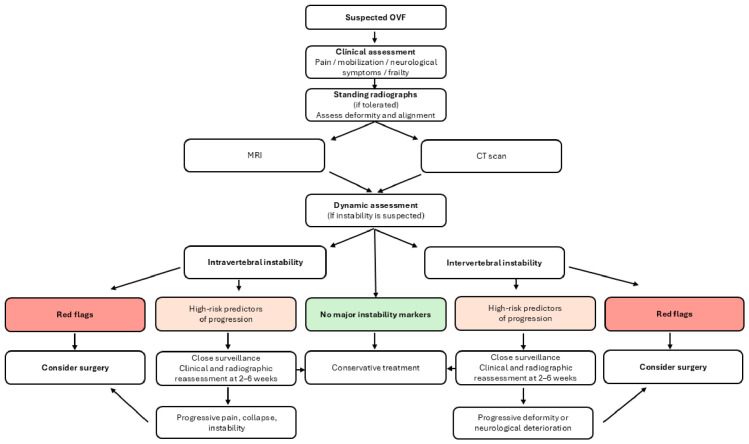
Proposed algorithm for instability stratification in osteoporotic vertebral fractures (OVFs). Initial evaluation should combine clinical assessment and standing radiographs when tolerated. MRI and CT should be considered complementary imaging modalities rather than stage-dependent examinations. Dynamic imaging may help identify reducibility and motion-dependent instability when instability is suspected. Instability markers are categorized into red flags, major predictors of progression, and absence of major instability markers, guiding consideration of surgery, close surveillance, or conservative treatment. This algorithm should be interpreted as a decision-support framework rather than a prescriptive treatment tool.

In this context, we propose the following algorithm based on the two patterns of instability and the radiological markers summarized in Table 1 and Table 2. It is intended to provide a structured framework to support clinical decision-making by integrating imaging findings, clinical presentation, and predictors of progression (Figure 6). Within this framework, instability markers are stratified into red flags, major predictors of progression, or absence of major instability markers, guiding consideration of surgical treatment, close surveillance, or conservative management. However, this algorithm should be regarded as a decision-support framework rather than a prescriptive treatment tool.

Persistent intractable mechanical back pain despite adequate conservative treatment may favor surgery intervention [9,26,33]. Painful kyphosis is another common indication; many authors suggest surgery for regional kyphosis > 30° [26,75], whereas others (e.g., Spiegl [33]) advocate a more restrictive threshold of >20°. Additional relative radiological indications include progressive loss of segmental height [26], significant canal compromise even in the absence of neurological deficit [25,26], and wedge-type collapse [66]. Nonunion with spinal canal compromise [25] and VBON/Kümmell disease beyond early stages [35] should also prompt consideration of surgical treatment. Finally, the AO Spine–DGOU OF classification can guide decision-making: OF4 and OF5 fractures generally favor surgery, while OF3 fractures typically require careful discussion and individualization. This limitation is exemplified by the hinge fracture, which is currently classified as an OF4 fracture but does not adequately reflect the severity of its impact on sagittal alignment (Figure 3 and Figure 4).

When surgery is indicated, its goals are to improve or recover neurological deficits, stabilize the fracture, and restore spinal alignment, thereby reducing pain and enabling early mobilization and functional recovery. A wide range of techniques, approaches, and strategies has been described, and there is still no consensus regarding the optimal procedure [46,74]. Management must therefore be individualized, taking into account patient-related factors (clinical condition, frailty, and pain severity), fracture-related factors (morphology and risk of further collapse), and treatment-related considerations, including the invasiveness of the procedure and the resources and expertise available at the treating center, as no single approach is universally applicable to all OVFs.

### 8.2. Preoperative Dynamic Assessment

**Figure 7:**
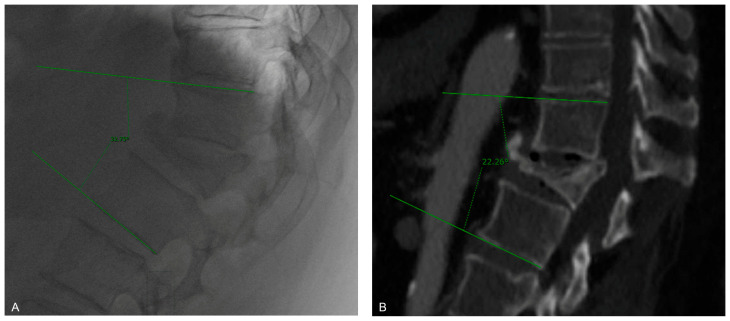
Regional kyphosis worsens in the standing position on radiographs (**A**) compared with the supine position on CT images (**B**).

An important preoperative step is the assessment of dynamic fracture mobility, as recommended by McKiernan [17]. Because OVFs are frequently associated with kyphosis and deformity, documenting reducibility is essential for surgical planning. Flexion–extension radiographs, or comparison of preoperative standing lateral radiographs with supine views can demonstrate reducibility and estimate potential kyphosis correction (Figure 7). The height of mobile fractures has been reported to increase by an average of 68% with positioning alone [17].

Guo et al. proposed an extended CT protocol to classify fractures as reducible or irreducible. Sagittal reconstructions allow for assessment of restoration of anterior vertebral height and changes in canal compromise [18]. Fractures are categorized as type 1 (reducible), with type 1.1 considered reducible and stable (one or two posterior wall fracture lines and intact pedicles) and type 1.2 considered reducible but unstable (≥3 posterior wall fracture lines and/or pedicle fracture and/or MRI evidence of PLC injury). Type 2 fractures are classified as irreducible, with a type 2M subgroup describing fractures that become reducible under anesthesia.

The rationale for this dynamic assessment is that a substantial degree of correction may be achieved with positioning alone. Therefore, evaluating the flexibility of the deformity is essential in surgical planning, as it may allow for adequate reduction without resorting to more aggressive procedures such as osteotomies [17].

### 8.3. Surgery and Instrumentation Present High Risks of Failure in OVF Population

Surgery and instrumentation carry a substantial risk of failure and complications in patients with OVF, largely because this population is frequently elderly, frail, and comorbid (e.g., restrictive pulmonary disease, cardiac conditions). In addition, many patients present with pre-existing spinal deformity (scoliosis, sagittal malalignment) and/or complex degenerative conditions such as diffuse idiopathic skeletal hyperostosis, ankylosing spondylitis, or other inflammatory disorders. These factors increase surgical complexity and narrow the margin for error.

From a technical standpoint, extensive constructs may be required to address instability or deformity, but long instrumentation increases morbidity and complication rates, and anterior approaches may be excessively invasive in fragile patients. Soft-tissue quality is also a concern: fragile skin and poor wound biology increase the risk of infection and impaired healing. In severe OVFs with neurological compromise, major reconstructive procedures may be necessary, potentially prolonging hospitalization and increasing the burden of perioperative complications, with a negative impact on postoperative quality of life [22].

Bone quality remains a central limitation. In osteoporotic bone, reduced implant fixation strength predisposes patients to screw loosening/pull-out and implant migration, making adequate fixation difficult to achieve [28]. Consistent with these challenges, overall complication rates have been reported to be high (e.g., 15.5% in some series [51]), and may be even higher when correction of sagittal malalignment requires long-segment instrumentation [76]. Mechanical complications such as proximal junctional kyphosis and distal junctional kyphosis are of particular concern. Finally, perioperative medical complications are common in this age group, including cardiac events, gastrointestinal bleeding, and postoperative cognitive disorders, and require specific attention in perioperative planning [49].

### 8.4. Towards Less Aggressivity

A wide spectrum of surgical strategies has been described for OVF, ranging from posterior decompression and fusion to anterior reconstruction aimed at restoring anterior column height [49], and corrective osteotomies to address kyphosis and sagittal malalignment [40,76,77]. However, given the frailty of many patients and the high complication burden associated with extensive reconstructions, the current trend is toward less invasive, tailored procedures, combining adapted fixation methods with minimally invasive (MIS) techniques to improve efficiency while limiting surgical morbidity.

Adapted implants and fixation strategies. In osteoporotic bone, reinforcement of pedicle screw fixation is frequently required to reduce loosening. Cement-augmented screws (typically using PMMA) are widely used because they provide a practical and effective means of increasing pull-out strength and improving construct stability [1,33,53]. Nevertheless, cement augmentation is associated with specific risks, including cement leakage and embolism, which must be anticipated and minimized through careful technique and patient selection. In the setting of anterior reconstruction, expandable cages and improved implant designs may help reduce surgical aggressiveness and limit cage subsidence by optimizing endplate contact and load distribution [78].

Minimally invasive techniques. MIS approaches—including percutaneous pedicle screw fixation and hybrid constructs combining vertebral augmentation (vertebroplasty/kyphoplasty) with percutaneous instrumentation—aim to reduce soft-tissue trauma, blood loss, and perioperative complications while maintaining sufficient stability [5]. In addition, less invasive osteotomy techniques have been described to decrease the risk of neurological injury and reduce operative bleeding compared with more aggressive corrective procedures [45,75,79].

## 9. Conclusions

Although most OVFs follow a benign course and can be managed conservatively, unstable fractures carry a substantial risk of adverse outcomes. Neurologic deficit represents the most severe complication. However, progressive vertebral collapse, kyphotic deformity, refractory pain, and severe sagittal malalignment may also occur, particularly in patients with multiple fractures, resulting in marked functional impairment and loss of autonomy.

As treatment decisions largely depend on the risk of progression, stability assessment constitutes a pivotal step in the management of OVF. In this review, we summarized the principal clinical and radiological markers as a structured framework. The distinction between intravertebral and intervertebral/regional instability provides a practical approach to localizing the underlying source of mechanical failure and to structuring the evaluation, thereby facilitating more rational and individualized treatment strategies. Rather than representing a binary state, instability in OVFs should be understood as a dynamic spectrum ranging from radiological predictors of progression to clear mechanical and neurological failure.

Classification systems may further support decision-making. In particular, the AO Spine–DGOU OF classification provides an OVF-specific framework that facilitates severity stratification and guide management. Nevertheless, given the considerable variability in fracture morphology, the presence of multiple fractures, bone quality, baseline deformity, comorbidities, and functional demands, treatment decisions must remain individualized. Surgical intervention is generally indicated in the presence of neurological compromise. In the absence of neurological deficits, relative indications typically include persistent pain, documented instability, progressive collapse, and/or progressive deformity with loss of sagittal balance.

An ideal surgical strategy should provide durable stability while minimizing morbidity, minimizing construct length when feasible, ensuring adequate fixation in osteoporotic bone, addressing deformity when clinically relevant, and reducing the risk of complications. However, in this elderly and often medically complex population, all techniques carry inherent limitations and potential complications. Ultimately, optimal outcomes depend on appropriate patient selection and a thorough, structured assessment of stability, integrating clinical status and imaging findings to determine the most suitable treatment for each individual patient.

## Data Availability

The original contributions presented in this study are included in the article/Supplementary Material. Further inquiries can be directed to the corresponding author.

## Supplementary Materials

The following supporting information can be downloaded at: https://www.mdpi.com/article/10.3390/diagnostics16121896/s1, Table S1: Key studies supporting instability markers and the proposed decision framework in osteoporotic vertebral fractures (OVFs).

## Abbreviations

The following abbreviations are used in this manuscript:

OVF	Osteoporotic Vertebral fractures
OF	Osteoporotic fractures
PLC	posterior ligamentous complex
IVC	intravertebral cleft
SVA	Sagittal Vertical Axis
AI	Artificial Intelligence
CSA	cross-sectional area
VBON	Vertebral Body necrosis
MIS	Minimally invasive techniques
